# Long‐term mental health outcomes after corneal transplantation and potential predictors: A multicentre prospective cohort study

**DOI:** 10.1111/opo.13432

**Published:** 2024-12-11

**Authors:** E. B. M. Elsman, H. P. A. Van der Aa, N. E. Billingy, C. Nieuwendaal, R. P. L. Wisse, R. J. Wijdh, M. L. Tang, B. T. H. Van Dooren, S. Nobacht, R. M. M. A. Nuijts, G. H. M. B. Van Rens, R. M. A. Van Nispen

**Affiliations:** ^1^ Ophthalmology Amsterdam UMC, Vrije Universiteit Amsterdam Amsterdam the Netherlands; ^2^ Amsterdam Public Health Research Institute Amsterdam the Netherlands; ^3^ Ophthalmology Amsterdam UMC, University of Amsterdam Amsterdam the Netherlands; ^4^ Ophthalmology University Medical Centre Utrecht Utrecht the Netherlands; ^5^ Ophthalmology University Medical Centre Groningen Groningen the Netherlands; ^6^ Ophthalmology Gelre Hospitals Apeldoorn the Netherlands; ^7^ Ophthalmology Amphia Hospital Breda the Netherlands; ^8^ Ophthalmology Radboud University Medical Centre Nijmegen the Netherlands; ^9^ Maastricht University Medical Centre+, University Eye Clinic Maastricht the Netherlands

**Keywords:** anxiety, corneal transplantation, depression, fatigue, mental health, patient‐reported outcomes

## Abstract

**Introduction:**

To evaluate the long‐term effect of corneal transplantation on mental health outcomes and to assess potential predictors of these outcomes.

**Methods:**

For this multicentre prospective cohort study, patients awaiting corneal transplantation were recruited from 11 (academic) hospitals and eye clinics in the Netherlands. Participants (*n* = 238) completed the Centre for Epidemiological Studies Depression scale (CES‐D), the Hospital Anxiety and Depression Scale‐Anxiety subscale (HADS‐A) and the Dutch ICF Activity Inventory Emotional Health subscale (DAI‐EH) and Fatigue subscale (DAI‐F) 1 month prior and 3, 6, 12 and 24 months after corneal transplantation. Sociodemographic and clinical characteristics, as well as coping styles as measured with the Utrecht Coping List, were considered as potential predictors for mental health outcomes (depression, anxiety, emotional health problems and fatigue). Linear mixed models were used to analyse and predict symptoms of depression, anxiety, emotional health problems and fatigue over time.

**Results:**

Scores on the CES‐D and HADS‐A improved significantly from baseline to 24‐months (mean scores CES‐D: 8.6 vs. 7.7, *p* = 0.03; mean scores HADS‐A: 3.7 vs. 3.2, *p* = 0.002). Scores on the DAI‐EH and DAI‐F also improved significantly from baseline to 24‐months (mean scores DAI‐EH: 10.7 vs. 7.5, *p* < 0.001; mean scores DAI‐F: 17.4 vs. 11.3, *p* < 0.001). Male sex and Fuchs' dystrophy were important predictors of better mental health outcomes, whereas comorbidity, (dry) eye complaints and a passive reacting coping style were important predictors of worse mental health outcomes.

**Conclusion:**

Corneal transplantation had a positive impact on mental health outcomes and important predictors were identified. This study may improve the understanding of patients and eyecare practitioners about the effects of corneal transplantation, leading to realistic communication about corneal transplantation expectations.

**Key points**

- Corneal transplantation significantly improves mental health outcomes, including symptoms of depression, anxiety, emotional health and fatigue over a 24‐month follow‐up period.
- Male sex, Fuchs' dystrophy and active coping styles predict better mental health outcomes, while comorbidity, dry eye complaints and passive coping styles are linked to poorer outcomes.
- Understanding these predictors can guide practitioners in providing more tailored preoperative counselling, potentially improving patient decision making and managing expectations regarding corneal transplantation.

## INTRODUCTION

Corneal diseases are a leading cause of visual impairment and blindness and affect approximately 4.9 million people worldwide.^1^, ^2^, ^3^, ^4^ Common corneal diseases include pseudophakic bullous keratopathy, keratoconus, trauma, keratitis, endothelial dysfunction and corneal stromal dystrophies. For advanced corneal diseases, corneal transplantation, which is the most frequently conducted type of transplantation, is the most effective intervention.^5^, ^6^ Over the past 20 years, corneal transplantation techniques have undergone considerable evolution, leading to a greater variety of keratoplasty techniques.^5^ Evaluations of postoperative outcomes have mainly focused on conventional clinical outcomes, such as visual acuity, astigmatism, endothelial count, graft clarity and complications. However, these clinical outcomes do not adequately capture the experience of the patient. Therefore, in recent years, more emphasis is being placed on patient‐reported outcomes after corneal transplantation^7^, ^8^, ^9^, ^10^, ^11^, ^12^ which capture how the patient is doing in terms of daily functioning and quality of life.

A systematic review evaluating the effects of corneal transplantation on patient‐reported outcomes, such as health/vision‐related quality of life, visual functioning, self‐reported visual symptoms and patient satisfaction showed overall beneficial effects.^9^ Predictors of positive patient‐reported outcomes were lower preoperative visual acuity and visual functioning, more favourable postoperative clinical outcomes, younger age and male sex. Most studies have focused on vision‐related or health‐related quality of life.^7^, ^13^, ^14^, ^15^, ^16^, ^17^, ^18^ Only limited research has been undertaken on mental health outcomes after corneal transplantation.^14^

It is well known that individuals with low vision are at risk for developing mental health complaints.^19^, ^20^ Previous studies have mainly focused on (older) adults with multiple causes of visual impairment, suggesting that approximately one‐third of visually impaired older adults experience mild but clinically significant symptoms of anxiety and/or depression.^20^ Drzyzga et al. evaluated the effects of corneal transplantation on mental health.^14^ This study showed that 16% of the patients awaiting corneal transplantation met the criteria for depression, while 20% met the criteria for anxiety. These numbers reduced significantly 3 weeks and 4 months after corneal transplantation, to 11% and 13% for both conditions at the two time points, respectively. However, the follow‐up period was short, and the sample size was relatively small. Moreover, not all important predictors of mental health outcomes, such as coping styles,^21^, ^22^ sociodemographic and clinical factors were taken into account. To better understand the impact of corneal transplantation and other predictors on mental health outcomes, this study aims to evaluate the effect of corneal transplantation on mental health outcomes and assesses potential predictors of these outcomes.

## METHODS

After reviewing the study protocol, study information letter and other documents, the Medical Ethical Committee of Amsterdam University Medical Centres (UMC), location VUmc, the Netherlands, confirmed that the study protocol was exempted from ethical approval according to the Dutch Medical Research in Human Subjects Act (WMO). The study adhered to the tenets of the Declaration of Helsinki. Written informed consent was obtained from all participants.

### Study design and setting

This multicentre prospective cohort study was conducted across 11 (academic) hospitals and eye clinics in the Netherlands: Amsterdam UMC (location VUMC and AMC), Maastricht UMC, UMC Utrecht, UMC Groningen, Leiden UMC, Radboud UMC, OMC (Ophthalmological Medical Centre) Zaandam, Deventer Hospital, Gelre Hospital Apeldoorn and Amphia Hospital Breda. Data were collected between September 2017 and January 2021.

### Participants and procedures

Patients were eligible to participate if they met the following inclusion criteria: age 18 years or older, awaiting corneal transplantation using lamellar or penetrating keratoplasty techniques, able to speak and understand the Dutch language and not having a severe cognitive impairment, as assessed by their attending ophthalmologist. Potential participants received a study information letter from their ophthalmologist. If they wanted to participate, patients returned the informed consent form to the researchers. Participants were subsequently telephoned by the researcher to explain the study procedures in more detail and to ask for the scheduled corneal transplantation date. One month prior to the corneal transplantation, participants received the baseline questionnaire (T0) through a web‐based survey platform. The option to complete questionnaires via paper‐and‐pencil versions or a telephone interview was offered to those not able to complete online questionnaires. Follow‐up questionnaires were sent 3 (T1), 6 (T2), 12 (T3) and 24 (T4) months after corneal transplantation. Clinical information such as corneal diagnosis (Fuchs' dystrophy, herpes, keratoconus, inflammation, other), the presence of other ocular diseases, use of medication, visual acuity, transplantation technique, tonometry, endothelial cell density and history of corneal transplantation were retrieved from patient records at the (academic) hospital or eye clinic.

### Outcome measures

The Centre for Epidemiological Studies Depression scale (CES‐D) was used to measure symptoms of depression. The CES‐D consists of 20 items with four response categories. Total scores range from 0 to 60 and higher scores are indicative of more depressive symptoms; a score of ≥16 indicates subthreshold depression.^23^ Anxiety symptoms were measured with the Hospital Anxiety and Depression Scale‐Anxiety subscale (HADS‐A). The HADS‐A consists of seven items with four response categories. Total scores range from 0 to 21 with higher scores representing more symptoms of anxiety; a score ≥8 indicates subthreshold anxiety.^24^, ^25^ Subthreshold depression and anxiety refer to a condition where individuals experience depressive or anxiety symptoms that are not severe enough to meet the full criteria for a clinical diagnosis (e.g., major depressive disorder or generalised anxiety disorder), but the symptoms cause noticeable stress or impairment.^26^ The Dutch ICF Activity Inventory Emotional Health subscale (DAI‐EH) and Fatigue subscale (DAI‐F) were used to measure dealing with emotional health problems and fatigue due to visual impairment, respectively.^27^, ^28^ The DAI‐EH consists of 14 items whereas the DAI‐F consists of nine items. All items have five response categories ranging from not difficult to impossible. A not applicable option is also available, and is treated as a missing value. Total scores on the DAI‐EH and DAI‐F were calculated on a scale from 0 to 100 if at least 75% of the items were answered, with higher scores representing more emotional health problems and fatigue, respectively. At baseline, scores of 15 participants on the DAI‐EH and two participants on the DAI‐F could not be calculated because of too many missing responses (i.e., <75% of the items were answered).

### Potential predictors

The following sociodemographic and clinical characteristics, for which participants completed questions at baseline (T0), were included as potential categorical predictors: age (four categories of similar size were created because of non‐linear relationship with outcomes: <65, 65–69, 70–74, 75+ years), sex, nationality (Dutch vs. non‐Dutch), living situation (alone vs. with others), work situation (no work vs. paid/voluntary work) and having comorbidity (either pulmonary diseases, cardiovascular diseases, gastrointestinal diseases, neurological diseases, diabetes, arthritis, cancer, psychiatric disorders and/or other health problems). Years of education was dichotomised into high (>10 years) vs. moderate–low (≤10 years) in the analyses of the DAI‐EH and DAI‐F because of a non‐linear relationship; for the CES‐D and HADS‐A, years of education was included as continuous predictor. Vision‐related predictors were primary diagnosis (Fuchs' dystrophy vs. other), former transplantation in the same eye, having a visual impairment (i.e., visual acuity ≥0.50 logMAR), transplantation technique (lamellar keratoplasty vs. penetrating keratoplasty) and occurrence of eye complaints. Occurrence of eye complaints was measured by the 10‐item Eye Complaint Questionnaire (ECQ), scored from 0 to 6 (score range 0–60)^29^ and the 8‐item Dry Eye Questionnaire (DEQ), scored from 0 to 3 (score range 0–24).^30^ Scores on the ECQ and DEQ were included as continuous predictors. Higher scores represented more (dry) eye complaints. Coping styles were measured with the Utrecht Coping List (UCL), consisting of 47 items scored from 1 to 4 which can be divided into seven subscales according to the scoring manual (three items are not assigned to any of the subscales and are therefore not used).^31^ The UCL is a widely used instrument which has been translated into several languages and is used in a range of populations. It demonstrated good measurement properties and was found to be feasible across diverse populations, including in large‐scale studies and clinical settings.^32^, ^33^, ^34^ Four subscales represent active coping styles: active tackling (seven items, score range 7–28), seeking social support (six items, score range 6–24), expressing emotions (three items, score range 3–12) and reassuring thoughts (five items, score range 5–20). The other three subscales represent passive coping styles: palliative reacting (eight items, score range 8–32), avoiding (eight items, score range 8–32) and passive reacting (seven items, score range 7–28). For all seven coping styles, participants' scores were dichotomised into ‘high’ scores (i.e., representing high or very high scores) and ‘low’ scores (i.e., representing average, low or very low scores) based on normative values.^31^

### Statistical analyses

Data were analysed using SPSS version 28 (ibm.com). Descriptive statistics were used for baseline characteristics. Independent sample *t*‐tests and chi‐square tests were used to evaluate potential differences between participants who completed the study and those who were lost to follow up. After checking relevant assumptions (e.g., normality, linearity and multicollinearity), linear mixed model analyses were performed to analyse and predict symptoms of depression, anxiety, emotional health problems and fatigue over time. First, univariable analyses were conducted to explore the relation between each predictor and the outcome; a *p*‐value <0.30 was used for selection in the multivariable model. Subsequently, multivariable linear mixed model analyses with backward stepwise selection were performed. A *p*‐value ≥0.05 was used to exclude predictors. Final models were validated using a shrinkage factor derived from the heuristic shrinkage estimate from van Houwelingen and le Cessie.^35^ Clinical significance of the findings was evaluated using Cohen's effect sizes, where 0.20–0.49 are considered small, 0.50–0.79 moderate and ≥0.80 large.^36^

## RESULTS

### Demographic and clinical patient characteristics

In total, 238 participants were included in the study. Their baseline (T0) sociodemographic and clinical characteristics are presented in Table 1. At T4, loss to follow‐up was 13.9% (*n* = 33). Participants who completed the study had a significantly higher education in years (mean 11.6 vs. 10.1, *p* = 0.008) and lower baseline scores on the CES‐D (mean 7.9 vs. 12.5, *p* < 0.001), HADS‐A (mean 3.5 vs. 4.9, *p* = 0.02) and DAI‐EH (mean 10.1 vs. 15.7, *p* = 0.01). There was a significant association between completing the study and nationality, having work (paid/voluntary) and having an ‘avoiding’ coping style: 88% of Dutch participants completed the study versus 62% of non‐Dutch participants (*p* = 0.008); 78% of participants scoring high on the ‘avoiding’ coping style completed the study versus 90% who scored low (*p* = 0.02) and 92% of participants with work completed the study versus 82% of participants without work (*p* = 0.045).

**TABLE 1:** Baseline (T0) sociodemographic and clinical characteristics of participants (*N* = 238)^a^.

Characteristic	
Age in years, mean (SD) [range]	67.6 (11.0) [24–87]
Female, *n* (%)	130 (54.6)
Education in years, mean (SD) [range]	11.4 (2.7), 6–16
Living alone, *n* (%)	60 (25.2)
Dutch nationality, *n* (%)	225 (94.5)
Comorbidity, *n* (%)	111 (47.0)
Work situation	
Paid work, *n* (%)	54 (22.8)
Voluntary work, *n* (%)	41 (17.3)
Primary diagnosis, *n* (%)	
Fuchs' dystrophy	137 (58.1)
Keratoconus	10 (4.2)
Herpes	6 (2.5)
Other	83 (35.2)
logMAR visual acuity of the best eye, mean (SD) [range]	0.31 (0.41), [−0.18–2.48]
logMAR visual acuity ≥0.50, *n* (%)	38 (17.4)
Former transplantation in same eye, *n* (%)	41 (17.4)
Transplantation technique, *n* (%)	
Lamellar keratoplasty	
Anterior	
DALK	5 (2.1)
Posterior	
DMEK	107 (45.5)
DSAEK	68 (28.9)
DSEK	5 (2.1)
PLK	8 (3.4)
Penetrating keratoplasty	
PK/PKP	42 (17.9)
Endothelial cell count donor, mean (SD) [range]	2698 (197) [1479–3100]
ECQ score, mean (SD) [range]	18.6 (10.8) [0–49]
DEQ score, mean (SD) [range]	8.8 (4.7) [0–21.7]
Scoring high on coping style, *n* (%)	
Active coping styles	
Active tackling	85 (36.2)
Seeking social support	82 (34.9)
Expressing emotions	42 (17.9)
Reassuring thoughts	83 (35.5)
Passive coping styles	
Palliative reacting	83 (35.3)
Avoiding	63 (26.8)
Passive reacting	22 (9.9)

Abbreviations: DALK, deep anterior lamellar keratoplasty; DEQ, Dry Eye Questionnaire; DMEK, Descemet membrane endothelial keratoplasty; DSAEK, Descemet‐stripping automated endothelial keratoplasty; DSEK, Descemet‐stripping endothelial keratoplasty; ECQ, Eye Complaint Questionnaire; PLK, posterior lamellar keratoplasty; PK/PKP, penetrating keratoplasty.

^a^ Most variables had missing values, ranging from *n* = 2–19; the percentages reported are the ‘valid percentages’ which exclude the missing values.

**FIGURE 1:**
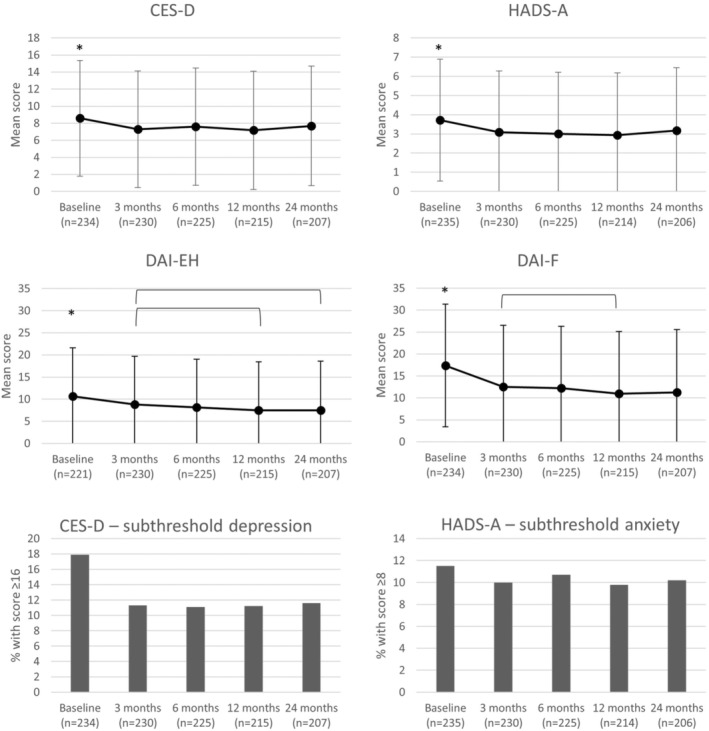
Depression (CES‐D), anxiety (HADS‐A), emotional health problems (DAI‐EH) and fatigue (DAI‐F) at baseline (T0) and 3 (T1), 6 (T2), 12 (T3) and 24 months (T4). The top four figures represent the mean score over time for the CES‐D, HADS‐A, DAI‐EH and DAI‐F; the bottom two figures represent the percentage of participants with scores indicative of subthreshold depression (CES‐D) and subthreshold anxiety (HADS‐A) over time. *Significant difference (*p* < 0.05) compared to all follow‐up measurements; brackets indicate significant differences (*p* < 0.05) between follow‐up measurements. CES‐D, Centre for Epidemiological Studies Depression scale; DAI‐EH, Dutch ICF Activity Inventory Emotional Health subscale; DAI‐F, Dutch ICF Activity Inventory Fatigue subscale; HADS‐A, Hospital Anxiety and Depression Scale‐Anxiety subscale.

Figure 1 shows mean scores of the CES‐D, HADS‐A, DAI‐EH and DAI‐F at baseline and follow‐up. Scores on the CES‐D and HADS‐A decreased between baseline and 3 months, after which time they stabilised. Scores on the DAI‐EH and DAI‐F decreased over time, but the largest decrease was observed between baseline and 3 months. Compared to baseline, scores at 24‐months follow‐up were significantly better for all outcomes (*p* < 0.05), but with small effect sizes (0.13 for CES‐D, 0.16 for HADS‐A, 0.29 for DAI‐EH and 0.43 for DAI‐F). At baseline, 42 participants (18%) were classified as having subthreshold depression, whereas 27 (12%) were classified as having subthreshold anxiety. The number of participants classifying as having subthreshold depression decreased between baseline and 3 months and stabilised thereafter, whereas the number of participants classifying as having subthreshold anxiety remained relatively stable over all measurements.

### Predictors of depression, anxiety, emotional health problems and fatigue

Table 2 shows the results of univariable and multivariable linear mixed model analyses for the CES‐D, HADS‐A, DAI‐EH and DAI‐F. The final model of the CES‐D showed that being 75 years or older, having comorbidity, having more (dry) eye complaints and having a ‘passive reacting’ and/or ‘expressing emotions’ coping style were significant predictors of more depressive symptoms. Male sex, education in years, having Fuchs' dystrophy and having an ‘active tackling’ coping style were significant predictors of lower levels of depressive symptoms. The HADS‐A final model showed that having comorbidity, having more (dry) eye complaints and having an ‘avoiding’ and/or ‘passive reacting’ coping style were significant predictors of more anxiety symptoms. Male sex, having Fuchs' dystrophy and having an ‘active tackling’ coping style were significant predictors of lower levels of anxiety. For the DAI‐EH, the final model showed that having comorbidity, more (dry) eye complaints and having a ‘passive reacting’ and/or ‘expressing emotions’ coping style were significant predictors of more emotional health problems. Male sex, having Fuchs' dystrophy and having an ‘active tackling’ coping style were significant predictors of less emotional health problems. The final model of the DAI‐F showed that living alone, having comorbidity, having more (dry) eye complaints and having a ‘passive reacting’ coping style were significant predictors of more fatigue. Male sex, having work and having Fuchs' dystrophy were significant predictors of less emotional health problems.

**TABLE 2:** Predictors of depression (CES‐D), anxiety (HADS‐A), emotional health problems (DAI‐EH) and fatigue (DAI‐F).

Predictor	Univariable analyses			Multivariable (full model)			Multivariable (final model)		
	Beta	95% CI	*p*‐Value	Beta	95% CI	*p*‐Value	Beta	95% CI	*p*‐Value
*CES‐D*									
CES‐D score^a^									
3 months	−1.28	−2.03; −0.53	<0.001	−0.19	−0.97; 0.60	0.64	−0.34	−1.10; 0.42	0.38
6 months	−0.98	−1.73; −0.22	0.01	−0.03	−0.84; 0.78	0.94	−0.17	−0.95; 0.60	0.66
12 months	−1.40	−2.17; −0.64	<0.001	−0.33	−1.16; 0.49	0.43	−0.33	−1.12; 0.46	0.42
24 months	−0.89	−1.66; −0.11	0.03	0.49	−0.34; 1.33	0.25	0.22	−0.58; 1.03	0.58
Male sex	−3.25	−4.73; −1.78	<0.001	−1.63	−2.76; −0.50	0.005	−1.81	−2.85; −0.77	<0.001
Age^b^									
65–69	−2.12	−4.24; −0.01	0.05	0.28	−1.28; 1.83	0.72	−0.00	−1.44; 1.43	1.00
70–74	0.15	−1.98; 2.28	0.89	1.14	−0.55; 2.82	0.19	1.36	−0.10; 2.82	0.07
75+	0.85	−1.26; 2.95	0.43	1.49	−0.35; 3.29	0.11	2.01	0.54; 3.48	0.008
Dutch nationality	−0.66	−4.12; 2.80	0.71						
Years of education	−0.39	−0.67; −0.11	0.01	−0.29	−0.50; −0.08	0.01	−0.27	−0.47; −0.08	<0.001
Living alone	3.01	1.30; 4.72	<0.001	1.04	−0.28; 2.38	0.12			
Having work (paid/voluntary)	−1.59	−3.13; −0.05	0.04	−0.14	−1.45; 1.16	0.45			
Comorbidity	1.97	0.46; 3.48	0.01	1.32	0.23; 2.42	0.02	1.44	0.42; 2.45	0.006
Fuchs' dystrophy	−3.07	−4.58; −1.57	<0.001	−2.42	−3.73; −1.17	<0.001	−2.51	−3.60; −1.46	<0.001
Former transplant	3.17	1.19; 5.14	0.002	1.07	−0.58; 2.74	0.20			
Visual impairment	1.00	−1.05; 3.05	0.34	−0.37	−1.93; 1.20	0.64			
Penetrating keratoplasty	2.17	0.16; 4.17	0.03	−1.16	−3.12; 0.81	0.25			
DEQ score	0.36	0.27; 0.45	<0.001	0.13	0.03; 0.23	0.01	0.14	0.05; 0.24	0.004
ECQ score	0.21	0.17; 0.25	<0.001	0.12	0.07; 0.17	<0.001	0.12	0.07; 0.16	<0.001
‘Active tackling’ coping style	−2.01	−2.78; −1.23	<0.001	−1.66	−2.41; −0.91	<0.001	−1.71	−2.42; −1.01	<0.001
‘Palliative reacting’ coping style	0.12	−0.62; 0.85	0.76						
‘Avoiding’ coping style	0.77	−0.02; 1.57	0.06	0.46	−0.31; 1.23	0.25			
‘Seeking social support’ coping style	−0.22	−0.99; 0.55	0.57						
‘Passive reacting’ coping style	8.34	7.13; 9.56	<0.001	7.39	6.12; 8.66	<0.001	7.38	6.17; 8.59	<0.001
‘Expressing emotions’ coping style	1.89	1.01; 2.76	<0.001	1.20	0.29; 2.10	0.009	1.18	0.33; 2.03	0.007
‘Reassuring thoughts’ coping style	−0.04	−0.83; 0.75	0.92						
*HADS‐A*									
HADS‐A score^a^									
3 months	−0.63	−0.95; −0.31	<0.001	−0.24	−0.58; 0.10	0.16	−0.25	−0.58; 0.07	0.13
6 months	−0.71	−1.03; −0.39	<0.001	−0.32	−0.66; 0.03	0.07	−0.31	−0.64; 0.03	0.08
12 months	−0.78	−1.10; −0.45	<0.001	−0.32	−0.67; 0.03	0.07	−0.32	−0.66; 0.02	0.06
24 months	−0.53	−0.86; −0.20	0.002	−0.15	−0.51; 0.21	0.40	−0.16	−0.51; 0.18	0.35
Male sex	−1.71	−2.42; −1.00	<0.001	−0.96	−1.55; −0.37	0.002	−1.09	−1.63; −0.55	<0.001
Age^b^									
65–69	−1.00	−2.03; 0.03	0.06	−0.03	−0.81; 0.76	0.95			
70–74	−0.23	−1.26; 0.81	0.67	0.34	−0.47; 1.15	0.41			
75+	−0.73	−1.75; 0.29	0.16	−0.07	−0.91; 0.78	0.87			
Dutch nationality	−0.02	−1.68; 1.64	0.98						
Years of education	−0.08	−0.22; 0.05	0.23	−0.07	−0.17; 0.04	0.22			
Living alone	0.63	−0.20; 1.47	0.14	0.04	−0.66; 0.74	0.90			
Having work (paid/voluntary)	−0.26	−1.00; 0.49	0.50						
Comorbidity	0.98	0.26;1.70	0.008	0.71	0.15; 1.27	0.01	0.68	0.15; 1.21	0.01
Fuchs' dystrophy	−1.10	−1.82; −0.36	0.004	−0.87	−1.54; −0.20	0.01	−0.94	−1.48; −0.40	<0.001
Former transplant	0.87	−0.09; 1.83	0.07	−0.15	−1.00; 0.70	0.73			
Visual impairment	0.47	−0.55; 1.49	0.37						
Penetrating keratoplasty	1.06	0.09; 2.03	0.03	0.28	−0.68; 1.24	0.56			
DEQ score	0.16	0.12; 0.20	<0.001	0.08	0.03; 0.12	<0.001	0.09	0.04; 0.12	<0.001
ECQ score	0.09	0.07; 0.10	<0.001	0.05	0.02; 0.07	<0.001	0.04	0.02; 0.06	<0.001
‘Active tackling’ coping style	−0.57	−0.91; −0.23	<0.001	−0.52	−0.85; −0.19	0.002	−0.48	−0.79; −0.17	0.003
‘Palliative reacting’ coping style	0.39	0.08; 0.71	0.01	0.21	−0.12; 0.55	0.21			
‘Avoiding’ coping style	0.44	0.10; 0.78	0.01	0.30	−0.04; 0.64	0.09	0.34	0.01; 0.67	0.04
‘Seeking social support’ coping style	0.08	−0.26; 0.41	0.66						
‘Passive reacting’ coping style	3.30	2.76; 3.84	<0.001	2.89	2.33; 3.45	<0.001	2.83	2.29; 3.37	<0.001
‘Expressing emotions’ coping style	0.53	0.15; 0.91	0.006	0.15	−0.24; 0.54	0.45			
‘Reassuring thoughts’ coping style	0.20	−0.15; 0.54	0.26	0.19	−0.16; 0.55	0.28			
*DAI‐EH*									
DAI‐EH score^a^									
3 months	−1.86	−2.96; −0.76	<0.001	0.16	−0.96; 1.28	0.78	0.33	−0.79; 1.45	0.57
6 months	−2.53	−3.64; −1.43	<0.001	−0.20	−1.36; 0.96	0.79	−0.11	−1.26; 1.03	0.84
12 months	−3.21	−4.33; −2.09	<0.001	−0.80	−1.97; 0.38	0.18	−0.60	−1.76; 0.57	0.32
24 months	−3.19	−4.32; −2.05	<0.001	−0.54	−1.73; 0.65	0.37	−0.47	−1.65; 0.72	0.44
Male sex	−4.71	7.15; −2.27	<0.001	−2.33	−4.17; −0.49	0.01	−2.68	−4.47; −0.90	0.003
Age^b^									
65–69	−5.34	−8.79; −1.89	0.003	−0.87	−3.36; 1.62	0.49			
70–74	−3.31	−6.79; 0.17	0.06	0.01	−2.49; 2.51	0.99			
75+	−1.72	−5.15; 1.71	0.32	0.73	−1.85; 3.31	0.58			
Dutch nationality	−0.10	5.74; 5.54	0.97						
Low–middle education	−0.10	−2.51; 2.31	0.93						
Living alone	3.36	0.50; 6.23	0.02	0.67	−1.49; 2.83	0.54			
Having work (paid/voluntary)	−1.32	−3.88; 1.24	0.31						
Comorbidity	3.78	1.29; 6.26	0.003	1.75	−0.03; 3.52	0.05	2.48	0.73; 4.23	0.006
Fuchs' dystrophy	−5.27	−7.73; −2.81	<0.001	−3.74	−5.84; −1.63	<0.001	−3.87	−5.65; −2.09	<0.001
Former transplant	5.22	1.95; 8.49	0.002	0.82	−1.83; 3.46	0.54			
Visual impairment	2.51	−0.60; 5.62	0.11	0.61	−1.91; 3.13	0.63			
Penetrating keratoplasty	3.93	0.66; 7.20	0.02	−2.80	−5.90; 0.30	0.08			
DEQ score	0.65	0.52; 0.78	<0.001	0.29	0.14; 0.44	<0.001	0.29	0.14; 0.43	<0.001
ECQ score	0.42	0.36; 0.48	<0.001	0.28	0.21; 0.35	<0.001	0.29	0.23; 0.36	<0.001
‘Active tackling’ coping style	−2.13	−3.28; −0.99	<0.001	−1.38	−2.47; −0.30	0.01	−1.70	−2.75; −0.64	0.002
‘Palliative reacting’ coping style	0.51	−0.57; 1.59	0.35						
‘Avoiding’ coping style	1.07	−0.09; 2.24	0.07	0.67	−0.42; 1.77	0.23			
‘Seeking social support’ coping style	−0.56	−1.69; 0.58	0.34						
‘Passive reacting’ coping style	9.83	7.95; 11.71	<0.001	7.82	6.01; 9.64	<0.001	7.75	5.95; 9.54	<0.001
‘Expressing emotions’ coping style	2.59	1.30; 3.87	<0.001	1.54	0.26; 2.82	0.02	1.82	0.57; 3.06	0.004
‘Reassuring thoughts’ coping style	0.41	−0.76; 1.59	0.49						
*DAI‐F*									
DAI‐F score^a^									
3 months	−4.84	−6.24; −3.45	<0.001	−1.57	−3.06; −0.09	0.04	−1.57	−2.98; −0.16	0.03
6 months	−5.20	−6.60; −3.79	<0.001	−1.31	−2.85; 0.22	0.09	−1.39	−2.83; 0.06	0.06
12 months	−6.43	−7.85; −5.00	<0.001	−2.44	−4.00; −0.88	0.002	−2.12	−3.59; −0.65	0.005
24 months	−6.13	−7.57; −4.69	<0.001	−1.78	−3.35; −0.20	0.03	−1.55	−3.04; −0.06	0.04
Male sex	−5.26	−8.44; −2.08	0.001	−2.16	−4.42; 0.11	0.06	−2.35	−4.51; −0.19	0.03
Age^b^									
65–69	−5.94	−10.41; −1.47	0.01	−1.06	−4.24; 2.12	0.51			
70–74	−3.62	−8.12; 0.88	0.11	−0.55	−3.91; 2.82	0.75			
75+	−0.29	−4.73; 4.16	0.90	1.70	−1.87; 5.27	0.35			
Dutch nationality	−4.84	−12.11; 2.42	0.19	−1.53	−6.79; 3.73	0.58			
Low–middle education	0.22	−3.01; 3.45	0.89						
Living alone	4.88	1.20; 8.57	0.01	1.04	−1.63; 3.72	0.44	2.93	0.47; 5.39	0.02
Having work (paid/voluntary)	−3.58	−6.86; −0.30	0.03	−2.57	−5.10; −0.05	0.05	−3.28	−5.40; −1.15	0.003
Comorbidity	6.34	3.18; 9.50	<0.001	4.24	2.03; 6.44	<0.001	4.46	2.36; 6.56	<0.001
Fuchs' dystrophy	−6.21	−9.40; −3.02	<0.001	−2.51	−5.10; 0.08	0.06	−3.63	−5.79; −1.47	0.001
Former transplant	6.86	2.65; 11.07	0.002	0.05	−3.21; 3.30	0.98			
Visual impairment	3.22	−0.85; 7.28	0.12	0.54	−2.56; 3.64	0.73			
Penetrating keratoplasty	6.85	2.68; 11.02	0.001	1.05	−2.87; 4.96	0.60			
DEQ score	1.08	0.92; 1.25	<0.001	0.55	0.96; 0.74	<0.001	0.55	0.37; 0.73	<0.001
ECQ score	0.66	0.59; 0.74	<0.001	0.47	0.38; 0.57	<0.001	0.50	0.41; 0.59	<0.001
‘Active tackling’ coping style	−1.48	−2.95; −0.01	0.05	−1.03	−2.46; 0.41	0.16			
‘Palliative reacting’ coping style	0.34	−1.05; 1.72	0.63						
‘Avoiding’ coping style	0.55	−0.95; 2.04	0.48						
‘Seeking social support’ coping style	−0.30	−1.76; 1.16	0.68						
‘Passive reacting’ coping style	9.14	6.63; 11.64	<0.001	5.86	4.47; 8.26	<0.001	5.68	3.41; 7.94	<0.001
‘Expressing emotions’ coping style	1.29	−0.37; 2.94	0.13	−0.42	−2.13; 1.28	0.63			
‘Reassuring thoughts’ coping style	−0.06	−1.56; 1.44	0.94						

Abbreviations: CES‐D, Centre for Epidemiological Studies Depression scale; DAI‐EH, Dutch ICF Activity Inventory Emotional Health subscale (DAI‐EH); DAI‐F, Dutch ICF Activity Inventory Fatigue subscale; DEQ, Dry Eye Questionnaire; ECQ, Eye Complaint Questionnaire; HADS‐A, Hospital Anxiety and Depression Scale‐Anxiety subscale.

^a^ Baseline was the reference category.

^b^ The age group <65 was the reference category.

### Internal validation of prediction models

The longitudinal models with only a random intercept at the participant level had higher −2 log likelihood (−2LL) values than the final prediction models. This indicates that the final models can explain more variance in the outcome measure than the models with only a random intercept. The CES‐D model with only a random intercept at the patient level had a −2LL of 6833, whereas the final model had a −2LL of 5757. The HADS‐A model with only a random intercept at the patient level had a −2LL of 5000, whereas the final model had a −2LL of 4426. The DAI‐EH model with only a random intercept at the patient level had a −2LL of 7645, whereas the final model had a −2LL of 6804. And the DAI‐F model with only a random intercept at the patient level had a −2LL of 8758, whereas the final model had a −2LL of 7367. Using the −2LL, the heuristic shrinkage estimate was calculated to be 0.99 for the CES‐D, 0.98 for the HADS‐A and 0.99 for both the DAI‐EH and DAI‐F, suggesting a potentially good calibration of the models in an external dataset.^35^, ^37^

## DISCUSSION

This study evaluated the effect of corneal transplantation on mental health outcomes and assessed potential predictors of these outcomes. This study found that depression and anxiety symptoms decreased immediately after corneal transplantation, as did the proportion of participants with subthreshold depression. Emotional health and fatigue outcomes also improved after corneal transplantation and continued to improve throughout the study. Important predictors of mental health outcomes were also identified.

A prevalence of 17.9% was found for subthreshold depression in this study, which is notably higher than the prevalence of 12% previously reported in a Dutch population of older adults.^20^ It is also higher than the 8.5% of adults aged 18–75 years with major depressive disorder in the Netherlands in 2019–2022.^38^ The prevalence of subthreshold anxiety was 11.5% in the present study, which is similar to the prevalence among Dutch older adults (10.7%).^20^ However, it is lower than the prevalence of anxiety disorders among adults aged 18–75 years in the Netherlands (15.2%).^38^ Yet, the numbers found here are lower than those observed in populations with visual impairment, where approximately one in three report having subthreshold depression and/or anxiety.^20^ If only participants with low vision from this study are considered (i.e., visual acuity ≥0.50 logMAR), then the percentages for subthreshold depression and anxiety at baseline increase to 21.6% and 13.5%, respectively.

Depression, anxiety, emotional health and fatigue scores improved significantly over time compared to baseline, yet effect sizes were small. The percentage of participants with subthreshold depression decreased from 17.9% at baseline to around 11% at each of the follow‐up measurements. The minimal clinically important difference (MCID) of the CES‐D is suggested to be around 11 points in a German sample with depression symptoms (as measured by the CES‐D‐15).^39^ In the present study, between baseline and 24‐months follow‐up, 13 participants (6%) improved at least 11 points, whereas between baseline and 3‐month follow‐up 14 participants (6%) improved at least 11 points. The percentage of participants with subthreshold anxiety decreased from 11.5% at baseline to around 10% at each of the follow‐up measurements. Although the MCID of the HADS‐A varies by population,^40^, ^41^, ^42^ an improvement of at least 3 points is often considered important. Between baseline and 24‐month follow‐up, 44 participants (21%) achieved this change, whereas between baseline and 3‐month follow‐up 41 participants (18%) achieved this change. No MCID values have been established for the DAI‐EH and DAI‐F. It is important to note that a large proportion of participants already had a (near) perfect score on these outcomes at baseline. Specifically, 50% of the participants scored ≤6 on the CES‐D, while 42% scored ≤2 on the HADS‐A (i.e., ≤10% of the maximum score). Additionally, 61% and 38% of the participants scored ≤10 on the DAI‐EH and DAI‐F, respectively. Hence, there was little room for improvement for these participants.

We found that male sex was a significant predictor of better scores in all four models, which is consistent with previous studies.^9^ Having the diagnosis Fuchs' dystrophy was also a significant predictor of better outcomes in all four models, whereas experiencing more (dry) eye complaints were significant predictors of worse scores in all four models. More than half of the participants in this sample had Fuchs' dystrophy, a common corneal disease that is often treated with the less invasive lamellar keratoplasty, which has a shorter recovery time. Other more severe diagnoses are often treated with penetrating keratoplasty, which requires a longer recovery period and may therefore lead to poorer mental health outcomes.^16^ However, transplantation technique was not a significant predictor in any of the models, and therefore other explanations might also play a role. For example, knowing that an effective and less invasive treatment is available, the gradual and slow progression of the condition,^43^ the relatively older age at which Fuchs' dystrophy becomes problematic and/or the isolation of the condition compared to corneal diseases that may be associated with systematic conditions may have a positive impact on mental health outcomes.

Exhibiting a passive reacting coping style was also a significant predictor of poorer outcomes in all models. This was the coping style least exhibited by participants in the current study. Yet, it largely influenced scores in each of the four models. People who exhibit this coping style allow themselves to become completely absorbed in their problems and the situation in which they find themselves. They often perceive their situation as gloomy and withdraw in worry, unable to do anything about their situation. This coping style is also recognised by worrying about the past.^31^ Exhibiting this coping style has been associated with poorer mental health.^31^, ^44^

Two other coping styles that have been associated with poorer psychological outcomes are the avoiding and expressing emotions coping styles.^31^, ^44^ The avoiding coping style was an additional significant predictor of poorer anxiety outcomes, and the expressing emotions coping style of poorer depression and emotional health outcomes. Although having a certain coping style has often been viewed as a stable trait of personality, studies have shown that therapy targeting specific coping strategies may be effective.^45^ This supports the view that the diverse coping strategies are not only a predictor of mental health outcomes but may also be a target for interventions aimed at improving mental health.

In contrast, the active tackling coping style was a significant predictor of better anxiety and emotional health outcomes. This coping style is characterised by a calm analysis of the situation so that the problem can be solved with confidence in a targeted manner. Active tackling is one of the active coping strategies and has previously been found to be a predictor of reduced symptoms of depression.^31^, ^44^, ^46^

We also found that living alone was a significant predictor of poorer fatigue scores as measured by the DAI‐F. Other studies have also noted that people who live alone report higher levels of fatigue.^47^, ^48^ On the other hand, having paid and/or voluntary work was a significant predictor of better fatigue scores. Individuals with higher levels of fatigue may find it more difficult to obtain or maintain employment, whether paid or voluntary. Fatigue can have a significant impact on various aspects of a person's life, including their ability to work effectively and cope with the demands of a job. A study in people with visual impairment found that fatigue had an impact on their cognitive functioning, particularly their concentration, attention and memory.^49^

Previous studies have shown that younger age is a predictor of more favourable patient‐reported outcomes, but no effect of age was found here, except for symptoms of depression as measured by the CES‐D. Participants aged 75 years or older had worse symptoms of depression compared with participants <65 years of age. The mean age of the present study population was 67.6 years, with poor representation of younger participants. Moreover, age had a non‐linear relationship with all outcomes, so age was categorised rather than including it as a continuous predictor. This may have resulted in loss of information.^50^, ^51^ Additionally, years of education was a significant predictor of better depression outcomes. The mean years of education in the sample was quite high, equivalent to at least a college degree. This may be due to selection bias, as highly educated people may be more willing to participate in research, while also reporting a higher quality of life.^52^

In contrast to previous studies,^9^ preoperative visual acuity was not a significant predictor in any of the four models. Participants were categorised as having visual impairment (i.e., visual acuity ≥0.50 logMAR) or no visual impairment because there was no linear relationship with any of the outcomes. Only 17.4% of participants had visual impairment at baseline, suggesting the sample size may have been too small to detect a significant effect. This finding may also be influenced by the current trends in transplantation indications. Nowadays, the indication for transplantation is more often given for higher vision than in the past, as people have higher requirements for vision, such as the desire to continue working. Consequently, they are more likely to need a transplant even with relatively better visual acuity. Additionally, the high success rate of transplantation may contribute to reduced mental complaints, as patients are optimistic about positive outcomes.

This study has several strengths. It is the first study to determine the long‐term effects of corneal transplantation on mental health outcomes. The sample size was large and high participation rates were maintained throughout the investigation. In addition, thanks to the collaboration of (academic) hospitals and eye clinics throughout the Netherlands, we were able to obtain a nationwide representation of participants.

Several limitations must also be acknowledged. First, the results of this study may not be generalisable to all patients undergoing corneal transplantation. Younger participants were poorly represented, although this is representative of the population undergoing corneal transplantation. In addition, the sample had a relatively high level of education. Thus, there is a risk of potential selection bias. This might have caused age, work status and years of education to be non‐significant predictors in most models. Also, the results may be less generalisable to other populations. Second, visual acuity was obtained from patient records at the (academic) hospital or eye clinic. However, this information was often poorly documented and most complete at baseline. Therefore, whether someone could be classified as visually impaired was based on the baseline visual acuity, and changes in visual acuity could not be taken into account. Furthermore, misclassification may have occurred because of poor documentation. Future studies should investigate whether changes in visual acuity act as a potential predictor of mental health outcomes. Third, no information was available regarding the use of immunosuppressive drugs after corneal transplantation, or information on primary graft failure. Information on previous transplantations in the same eye was available, which was not a significant predictor for any of the outcomes. Fourth, although a large number of potential predictors were included, other factors, such as perceived health status, may also play a role.^53^ Finally, we opted to use raw ordinal scores instead of response pattern scoring through item response theory (IRT) or Rasch analysis. Although we recognise the advantages that IRT/Rasch‐based scoring might offer, particularly with respect to missing data (which was the case for the DAI subscales), several considerations led us to use raw ordinal scores instead. These considerations included: (1) the unavailability of a scoring system (e.g., Excel (Microsoft.com) templates), which would require the development of entirely new item parameters, (2) the relatively small sample size in terms of IRT analyses (at least 500 participants are needed to adequately fit an IRT model^54^), affecting the ability to yield accurate parameter estimates and (3) the interpretability of raw scores, which can be more easily understood by a broader audience. Moreover, the CES‐D and HADS‐A are usually scored through raw ordinal scores, and cut‐off values for raw scores have previously been established. Thus, the scoring of the DAI subscales was consistent with the scoring of the CES‐D and HADS‐A.

In conclusion, this study shows that corneal transplantation has a positive impact on mental health outcomes, including depression symptoms, anxiety symptoms, emotional health problems and fatigue. Male sex and Fuchs' dystrophy were important predictors of better mental health outcomes, whereas having comorbidity, experiencing more (dry) eye complaints and having a passive reacting coping style were important predictors of worse mental health outcomes. The results of this study may improve the understanding of patients and eyecare providers regarding the effects of corneal transplantation. Practitioners could incorporate knowledge of predictors into preoperative counselling, which may lead to better decision making and more realistic patient expectations. Future studies are needed to confirm the results of this study, especially in underrepresented patient groups such as younger patients or those with lower education levels.

## AUTHOR CONTRIBUTIONS

**E. B. M. Elsman:** Data curation (equal); formal analysis (equal); investigation (equal); methodology (equal); project administration (equal); visualization (equal); writing – original draft (equal). **H. P. A. Van der Aa:** Supervision (equal); writing – review and editing (equal). **N. E. Billingy:** Data curation (equal); project administration (equal); writing – review and editing (equal). **C. Nieuwendaal:** Data curation (equal); writing – review and editing (equal). **R. P. L. Wisse:** Data curation (equal); writing – review and editing (equal). **R. J. Wijdh:** Data curation (equal); writing – review and editing (equal). **M. L. Tang:** Data curation (equal); writing – review and editing (equal). **B. T. H. Van Dooren:** Data curation (equal); writing – review and editing (equal). **S. Nobacht:** Data curation (equal); writing – review and editing (equal). **R. M. M. A. Nuijts:** Conceptualization (equal); data curation (equal); funding acquisition (equal); writing – review and editing (equal). **G. H. M. B. Van Rens:** Conceptualization (equal); funding acquisition (equal); supervision (equal); writing – review and editing (equal). **R. M. A. Van Nispen:** Conceptualization (equal); funding acquisition (equal); resources (equal); software (equal); supervision (equal); validation (equal); writing – review and editing (equal).

## FUNDING INFORMATION

The research is funded by Stichting Blindenhulp, Landelijke Stichting voor Blinden en Slechtzienden (LSBS), Hoornvlies Patiënten Vereniging, Katholieke Stichting voor Blinden en Slechtzienden (KSBS), Stichting tot Verbetering van het Lot der Blinden and Vereniging Bartiméus Sonneheerdt (VBS).

## CONFLICT OF INTEREST STATEMENT

The authors have no conflicts of interest.
